# Bayesian Model Selection Maps for Group Studies Using M/EEG Data

**DOI:** 10.3389/fnins.2018.00598

**Published:** 2018-09-28

**Authors:** Clare D. Harris, Elise G. Rowe, Roshini Randeniya, Marta I. Garrido

**Affiliations:** ^1^Computational Cognitive Neuroscience Laboratory, Queensland Brain Institute, The University of Queensland, Brisbane, QLD, Australia; ^2^Monash Neuroscience of Consciousness Laboratory, School of Psychological Sciences, Faculty of Medicine Nursing and Health Science, Monash University, Melbourne, VIC, Australia; ^3^School of Mathematics and Physics, The University of Queensland, Brisbane, QLD, Australia; ^4^Australian Research Council Centre of Excellence for Integrative Brain Function, Monash University, Melbourne, VIC, Australia; ^5^Centre for Advanced Imaging, The University of Queensland, Brisbane, QLD, Australia

**Keywords:** EEG, MEG, Bayes, PPMs, BMS, code:matlab, code:spm

## Abstract

Predictive coding postulates that we make (top-down) predictions about the world and that we continuously compare incoming (bottom-up) sensory information with these predictions, in order to update our models and perception so as to better reflect reality. That is, our so-called “Bayesian brains” continuously create and update generative models of the world, inferring (hidden) causes from (sensory) consequences. Neuroimaging datasets enable the detailed investigation of such modeling and updating processes, and these datasets can themselves be analyzed with Bayesian approaches. These offer methodological advantages over classical statistics. Specifically, any number of models can be compared, the models need not be nested, and the “null model” can be accepted (rather than only failing to be rejected as in frequentist inference). This methodological paper explains how to construct posterior probability maps (PPMs) for Bayesian Model Selection (BMS) at the group level using electroencephalography (EEG) or magnetoencephalography (MEG) data. The method has only recently been used for EEG data, after originally being developed and applied in the context of functional magnetic resonance imaging (fMRI) analysis. Here, we describe how this method can be adapted for EEG using the Statistical Parametric Mapping (SPM) software package for MATLAB. The method enables the comparison of an arbitrary number of hypotheses (or explanations for observed responses), at each and every voxel in the brain (source level) and/or in the scalp-time volume (scalp level), both within participants and at the group level. The method is illustrated here using mismatch negativity (MMN) data from a group of participants performing an audio-spatial oddball attention task. All data and code are provided in keeping with the Open Science movement. In doing so, we hope to enable others in the field of M/EEG to implement our methods so as to address their own questions of interest.

## Introduction

The statistical testing of hypotheses originated with Thomas Bayes (Neyman and Pearson, 1933), whose famous eponymous theorem (Bayes and Price, 1763) can be written in terms of probability densities as follows:

(1) $$p(\theta |y)=\frac{p(y|\theta )p(\theta )}{p(y)}$$

where *θ* denotes unobserved parameters, *y* denotes observed quantities, and *p*(θ|*y*) denotes the probability *p* of the unknown parameters θ, given (“|”) the set of observed quantities *y*. More generally, *p*(event|knowledge) denotes the probability of an event given existing knowledge. In other words, Bayes conceptualizes statistics as simply the plausibility of a hypothesis given the knowledge available (Meinert, 2012).

Bayes’ theorem allows one to update one’s knowledge of the previously estimated (or “prior”) probability of causes, to a new estimate, the “posterior” probability of possible causes. This process can be repeated indefinitely, with the prior being recursively updated to the new posterior each time. This gives rise to multiple intuitive and useful data analysis methods, one of which is the explained in detail in this paper.

Even when it first appeared, Bayes’ theorem was recognized as an expression of “common sense,” a “foundation for all reasonings concerning past facts,” (Bayes and Price, 1763). Centuries later, neuroscientific evidence suggests that Bayes’ theorem may not only explain our “common sense” and internal reasoning processes, but may be common to all our senses: it can actually explain the way in which we use our various senses to perceive the world. That is, Bayesian statistics can be used to accurately model and predict the ways in which our own brains process information (Dayan et al., 1995; Feldman and Friston, 2010; Friston, 2012; Hohwy, 2013). This has given rise to the concepts of predictive coding and the Bayesian brain. In this context, it is unsurprising that Bayesian approaches to statistics have high face validity (Friston and Penny, 2003). This allows for intuitive descriptions of probability and enables experimental results to be relatively easily understood and communicated both within and between scientific communities, as well as to the general public (Dunson, 2001).

Despite the intuitiveness of Bayesian approaches, however, the mainstay of hypothesis-testing since the 20th century (Vallverdú, 2008) has instead been classical or frequentist statistics, which conceptualizes probability as a “long-run frequency” of events, and which has dominated most approaches to neuroimaging analysis to date (Penny et al., 2003). For example, creating statistical parametric maps (SPMs), which is a popular method of analyzing neuroimaging data, mainly involves frequentist approaches (Friston and Penny, 2003).

In frequentist statistics, the null hypothesis (that there is no relationship between the causes and the data) is compared with one alternative hypothesis; the null is then either rejected in favor of the alternative hypothesis, or it fails to be rejected – it can never be directly “supported.” Rejection of the null depends on the somewhat unintuitive *p*-value, which communicates how likely it is that the effect (of at least the size seen in the experiment), would be seen in the absence of a true effect, if the experiment were repeated many times. This is a more complex and counterintuitive way of communicating results compared to Bayesian statistics (where the probability of the hypothesis in question is what is being estimated and communicated).

Also, unfortunately, multiple different models cannot be compared at once, and either the null and the alternative models need to be nested, or specific modifications need to be made (Horn, 1987; McAleer, 1995), for frequentist statistical tests to be feasible (Rosa et al., 2010). These features cause frequentist statistics to be less useful in certain contexts, compared to the approaches enabled by Bayesian statistics.

In recent decades, Bayesian approaches are becoming increasingly recognized for their superior utility for addressing certain questions and in specific data analysis situations, as explained below (Beal, 2003; Rosa et al., 2010; Penny and Ridgway, 2013). Importantly, with Bayesian approaches to data analysis, any number of models can be compared, the models need not be nested, and the “null model” can be accepted (Rosa et al., 2010). The fact that Bayesian hypothesis-testing also allows researchers to evaluate the likelihood of the null hypothesis is crucially important in light of the replication crisis in psychology and neuroscience (Hartshorne, 2012; Larson and Carbine, 2017; Szucs et al., 2017). Importantly, results supporting the null hypothesis are equally noteworthy or reportable as other results within Bayesian statistics. The use of Bayesian statistics may also ameliorate some statistical power-related problems documented in the literature (Dienes, 2016).

Even though Bayesian statistics has gained popularity in the context of “accepting the null,” its strength lies beyond this, in the sense that it enables the relative quantification of any number of *alternative* models (or hypotheses). In Bayesian Model Selection (BMS), models are compared based on the probability of observing a particular dataset given each model’s parameters. The probability of obtaining observed data, *y*, given model *m*, *p(y|m)*, is known as the model evidence. In BMS, an approximation of the model evidence is calculated for multiple models; the model evidences are then compared to determine which model returns the highest probability of generating the particular dataset in question (Rosa et al., 2010).

A computationally efficient and relatively accurate (Stephan et al., 2009) method of approximating the model evidence is to use variational Bayes (VB). If each participant in the dataset is assumed to have the same model explaining their data, then this is called a fixed effects (FFX) approach. If, on the other hand, every participant is permitted to have their own (potentially different) model, this is called a random effects (RFX) approach.

An elegant approach to succinctly communicating results is to use Posterior Probability Maps (PPMs), which provide a visual depiction of the spatial and/or temporal locations in which a particular model is more probable than the alternatives considered, given the experimental data in question. The development of PPMs is essentially the Bayesian alternative to the creation of SPMs (Friston and Penny, 2003). PPMs may display the posterior probability of the models (the probability that a model explains the data), or, alternatively, they may be displayed as Exceedance Probability Maps (EPMs), which are maps of the probabilities that a model (say *k)* is *more* likely compared to all other (*K*) models considered (Rosa et al., 2010). (EPMs will be identical to PPMs in cases where there are only two models being considered, as in this study.) EPMs are useful in that they allow us to directly quantify which model is more probable than the other/s considered.

The data analysis method that forms the focus of this paper is Posterior Probability Mapping with an RFX approach to VB. First introduced (Rosa et al., 2010) for functional magnetic resonance imaging (fMRI), the method has recently been adapted for inference using electroencephalography (EEG) data (Garrido et al., 2018). In their study, Garrido et al. (2018) used VB to approximate the log of the model evidence for each voxel (in space and time) in every participant, in order to construct PPMs at the group level. They did this in the context of comparing between two computational models describing the relationship between attention and prediction in auditory processing. While that paper focused on using this Bayesian methodology to address an important neuroscientific question, the precise way in which Rosa and colleagues’ (2010) methods were adapted for use with EEG data, has not been formally described to date – leading to the purpose of this paper.

Here, we describe in a tutorial-like manner how to build and compare PPMs for EEG and/or magnetoencephalography (MEG) data (M/EEG), using an RFX approach to VB. This approach provides useful ways of displaying the probabilities of different models at different times and brain locations, given any set of neuroimaging data [as done in Garrido et al. (2018)] using the Statistical Parametric Mapping (SPM) software package for MATLAB. Furthermore, in keeping with the Open Science movement, we provide the full EEG dataset^1^ and the code^2^ to facilitate future use of the method. In doing so, we hope that this paper and its associated scripts will enable others in the field of M/EEG to implement our methods to address their own questions of interest.

## Theory

In frequentist hypothesis testing, what is actually being tested is the null hypothesis (i.e., that there is no relationship between the variables of interest; Friston and Penny, 2007). If it is assumed that there is a linear relationship between the causes and data, then the relationship between the causes (*x*) and data (*y*) can be represented as below (Friston and Penny, 2007):

(2) y=xθ+ε

where *y* denotes data, *x* denotes causes and 𝜀 is an error term. The null hypothesis is that the relationship between the causes and data does not exist, that is*, θ* = 0. The null hypothesis is compared to one alternative hypothesis; the null is then either rejected in favor of the alternative hypothesis, or it fails to be rejected – it can never be directly “supported.”

Using the frequentist framework, one cannot test multiple models at once (unlike what can be done when using Bayesian approaches). (In this setting, a model corresponds to a particular mixture of explanatory variables in the design matrix *x*.) Even if one only wishes to test one model against the null, however, frequentist statistics still gives rise to problems unless the null and alternate models are nested. When the variables in one model cannot be expressed as a linear combination of the variables in another model, the two models are said to be non-nested (McAleer, 1995). Non-nested models usually arise when model specifications are subject to differences in their auxiliary assumptions or in their theoretical approaches, and can still be dealt with by making specific modifications to frequentist approaches (Horn, 1987; McAleer, 1995). However, there are many situations where Bayesian approaches are more appropriate for non-nested models than adapted frequentist inference (Rosa et al., 2010). Indeed, Penny et al. (2007a), showed that fMRI haemodynamic basis sets are best compared using Bayesian approaches to non-nested models.

Furthermore, Bayesian approaches to statistics have long been recognized for their relative advantages outside of the realm of neuroimaging. In clinical trials, Bayesian experimental design techniques and interim analyses have been found to improve trials’ statistical power, cost-effectiveness and clinical outcomes (e.g., Trippa et al., 2012; Connor et al., 2013), compared to when classical approaches are used alone. Bayesian statistics are also especially useful in the worlds of computational physics (Mohammad-Djafari, 2002) and biology (Needham et al., 2007), and in machine learning (Lappalainen and Miskin, 2000).

The aim of BMS is to adjudicate between models using each one’s *model evidence*. Also written as *p*(*y*|*m*), the model evidence is defined as the probability (*p*) of obtaining observed data (denoted *y*) given the model (denoted *m*). It is given by the following integral (Rosa et al., 2010):

(3) p(y|m)=∫p(y|θ, m)p(θ|m)dθ

This integral is usually intractable, so numerous methods have been developed to approximate it. As Blei et al. (2017) succinctly summarize, there are two main ways to solve the problem of approximating the integral above. One is to sample a Markov chain (Blei et al., 2017), and the other is to use optimisation. The conversion of an integration problem into an optimisation problem is due to Richard Feynman, who introduced variational free energy in the setting of path integral problems in quantum electrodynamics (Feynman and Brown, 1942; Feynman et al., 2010). By inducing a bound on the integral above – through an approximate posterior density (please see below) – one converts an intractable integration problem into a relatively straightforward optimisation problem, that can be solved using gradient descent.

Some of the specific approximation methods that have been used to date include Annealed Importance Sampling (AIS; Neal, 1998; Penny and Sengupta, 2016), Bayesian Information Criterion (BIC) measures (Rissanen, 1978; Schwarz, 1978; Penny, 2012), Akaike Information Criterion (AIC) measures (Akaike, 1980; Penny, 2012), and finally, the variational Free Energy (F), which was first applied to the analysis of functional neuroimaging time series by Penny et al. (2003) and which is explained in this paper (Rosa et al., 2010). These methods have varying degrees of accuracy and computational complexity, and have been studied in detail elsewhere (Beal and Ghahramani, 2003; Penny et al., 2004; Penny, 2012). The variational Free Energy provides a relatively high level of accuracy, without a great computational cost (Rosa et al., 2010), and so it is unsurprising that it is widely used in neuroimaging (Rosa et al., 2010). The Free Energy formula is (Penny et al., 2003):

(4) $$F=\int q(\theta |y)log\frac{p(y,\theta )}{q(\theta |y)}d\theta$$

where *q*(θ|*y*) is an (initially) arbitrary distribution of the parameters θ given the data at each voxel *y, p*(*y*,θ) denotes the joint probability of the data and the parameters occurring, and dθ simply denotes that the integral given by F is with respect to the model parameters θ.

The “variational” term in variational Free Energy, and in VB, refers to the branch of calculus (the calculus of variations) that deals with maximizing or minimizing functionals, or integrals. The utility of variational calculus in neuroimaging analysis has been reviewed in numerous other papers (Friston K.J. et al., 2008). In brief, the aim in VB is to maximize the functional given by the equation above. The reason for doing this is that it provides information about the model evidence. More specifically, the Free Energy relates to the log of the model evidence (or log-model evidence) as described by the following equation (Rosa et al., 2010), known as the fundamental equation (Penny et al., 2003) of Variational Bayes:

(5) logp(y|m)=F(m)+KL(q(θ)||p(θ|y,m))

where log *p*(*y*|*m*) is the log-model evidence, *F* is the variational Free Energy, and *KL*(*q*(θ)||*p*(θ|*y*,*m*)) is the Kullback–Leibler divergence (Kullback and Leibler, 1951), or relative information, with respect to the approximate distribution *q*(θ) and the distribution that is diverging from it, namely the true distribution, *p*(*θ|y*,*m*), as further described below.

The reason why Free Energy can be used as an approximation of the model evidence is better understood in light of the meaning of the second term in the fundamental VB equation, the Kullback–Leibler (KL) divergence (Penny et al., 2003). The equation for this is:

(6) $$KL=\int q(\theta |y\text{) }log\frac{q(\theta |y)}{p(\theta |y)}d\theta$$

where all terms listed here have the same meanings as defined in earlier paragraphs. The KL divergence is also known as KL information, and this is because it is a measure of the information “difference” or divergence between two distributions. It can be derived by considering the so-called cross-entropy and entropy of the two distributions, respectively, as outlined below (Carter, 2007). The concept of “relative entropy” is essentially “average information,” with “information” being defined as Shannon (1948/2001) originally introduced:

(7) $$I(p)=logb\left(\frac{1}{p}\right)=-logb\;(p)$$

where *I*(*p*) is the information given by observation of an event of probability *p*, and log_b_ (^1^/*_p_*) is the logarithm (in base *b*) of the inverse of the probability of that event. The formula above is used to derive the “average information,” also sometimes referred to as relative entropy, from a set of events. A related concept is the “cross entropy” between two distributions (see Carter, 2007); and the difference between the cross entropy and the entropy of the original/true distribution is equivalent to the KL divergence. Being a measure of information, the KL divergence has the property that it is non-negative; consequently, the lowest value it can take is zero.

The KL divergence between two distributions is zero only if the two distributions are equivalent. The closer KL is to zero, the less dissimilar the two distributions are. Thus, minimizing KL is equivalent to maximizing F, and F is said to provide a lower bound on the log-evidence. The aim of VB learning is to maximize F so that the approximate posterior thereby becomes as close as possible to the true posterior (Penny et al., 2007a).

If (and only if) the KL divergence is zero, then F is equal to the log-model evidence. The free energy thus provides a *lower bound* on the log-evidence of the model, which is why iteratively optimizing it allows us to proceed with BMS using F as an *approximation* of the log-model evidence (Penny et al., 2007a). As the KL divergence is minimized by an iterative process of optimisation, F becomes an increasingly “tighter” lower bound on the desired (actual) log-model evidence; owing to this, BMS can proceed using F as a “surrogate” for the log-model evidence (Rosa et al., 2010). The iterations continue until improvements in F are very small (below some desired threshold). This method of estimating the log-model evidence is implemented in the second script described in the Implementation section (“BMS2_ModelSpec_VB.m”).

Although it has been summarized here, it is also worth noting that VB is further fleshed out in multiple other research papers (Penny et al., 2003, 2007a; Friston et al., 2007) and tutorials (Lappalainen and Miskin, 2000). In *Statistical Parametric Mapping*, Friston (2007) provides the mathematical derivations for the fundamental equation of VB, and his colleagues provide a full explanation of its application to BMS (Penny et al., 2007b).

The application of VB in the context of fMRI analysis has been described in detail elsewhere (Penny et al., 2007a; Stephan et al., 2009; Rosa et al., 2010). Penny et al. (2007a) used Bayesian spatiotemporal models of within-subject log-model evidence maps for fMRI data, in order to make voxel-wise comparison of these maps and thereby to make inferences about regionally specific effects. Rosa et al. (2010) developed their approach by combining the methods described by Penny et al. (2007a) with those of Stephan et al. (2009), who used an RFX approach to VB, as described below.

After the log-model evidence has been estimated as described above, given uniform priors over models, one can then estimate posterior model probabilities by comparing model-evidences between models. The ratio between model evidences, or Bayes factor (BF), can be used to estimate posterior model probabilities. A BF greater than 20 is equivalent to a posterior model probability greater than 0.95 (Kass and Raftery, 1995), which is reminiscent of the typical *p*-value smaller than 0.05. The product of Bayes factors over all subjects is called the Group Bayes Factor (GBF), and it gives the relative probability that one model (relative to another) applies to the entire group of subjects. That is, it rests on the assumption that the data were generated by the same model for all participants, and that data are conditionally independent over subjects. This is known as fixed effects (FFX) inference, and it is not as robust to outliers as RFX inference, which does not assume that the data were necessarily generated by the same model for each participant (Stephan et al., 2009).

Stephan et al. (2009) developed a novel VB approach for group level methods of Bayesian model comparison that used RFX instead of fixed effects analysis at the group level. They did this by treating models as random variables whose probabilities can be described by a Dirichlet distribution (which is conjugate to the multinomial distribution) with parameters that are estimated using the log-model evidences over all models and subjects (as described below). Once the optimal Dirichlet parameters have been estimated, they can be used to calculate posterior probabilities or exceedance probabilities of a given model for a randomly selected participant. This is what is done in the third script (“BMS3_PPMs.m,” described in the Implementation section below), and the underlying mathematics is explained briefly below.

In the RFX approach introduced by Stephan et al. (2009), we assume that the probabilities of the different models (or hypotheses) are described by the following Dirichlet distribution:

(8) $$\begin{array}{c} p(r|\alpha )=Dir(r,\alpha )=\frac{1}{Z(\alpha )}\prod_{k}r_{k}^{\alpha_{k}-1}\\ Z(\alpha )=\frac{\prod_{k}\Gamma (\alpha_{k})}{\Gamma \left(\sum_{k}\alpha_{k}\right)} \end{array}$$

where *r* represents the probabilities *r* = [*r*_1_, …., *r*_K_] of *K* different models (or hypotheses), and α = [α_1,_ …., α_k_] are related to unobserved “occurrences” of models in the population. This distribution is part of a hierarchical model: the next level depends on model probabilities, *r*, which are described by the Dirichlet distribution.

In the next level of the hierarchical model, we assume that the probability that a particular model generated the data of a particular subject, is given by a multinomial variable *m_n_* whose probability distribution is as follows:

(9) $$p(m_{n}|r)=\prod_{k}r^{m_{nk}}$$

where m_n_ is the multinomial variable that describes the probability that model *k* generated the data of subject *n* given the probabilities *r*.

Finally, in the lowest level of this hierarchical model, the probability of the data in the *n*th subject, given model *k*, over all parameters (𝜗) of the selected model (i.e., the marginal likelihood of the data in the *n*th subject, obtained by integrating over the parameters of the model) is given by:

(10) $$p(y_{n}|m_{nk})=\int p(y|\vartheta )p(\vartheta |m_{nk})d\vartheta$$

The goal is to invert this hierarchical model, that is, work backward from data (*y*_n_) to find the parameters of the Dirichlet distribution (which then allows the calculation of the expected posterior probability of obtaining the *k*th model for any randomly selected subject, as shown below). This model inversion is done using a VB approach in which the Dirichlet distribution is approximated with a conditional density, *q(r)= Dir(*r*, α)*. Stephan et al. (2009) show that the following algorithm yields the optimal parameters of the conditional density *q(r)= Dir(*r*, α)*:

$$\alpha =\alpha_{0}$$

Until convergence

(11) $$\begin{array}{l} \begin{array}{l} u_{nk}=\exp\left(\ln p(y_{n}|m_{nk})+\psi (\alpha_{k})-\psi \left(\sum_{k}\alpha_{k}\right)\right)\\ \beta_{k}=\sum_{n}\frac{u_{nk}}{\sum_{k}u_{nk}}\\ \alpha =\alpha_{0}+\beta \end{array} \end{array}$$

where α are “occurrences” of models in the population; α_0_ is the Dirichlet prior, which, on the assumption that no models have been “seen” *a priori*, is set as α_0_ = [1,...,1] so that all models are equally probable to begin with; *u_nk_* is the non-normalized belief that model *k* generated the data *y_n_* for subject *n* (for the derivation of this line, please see Stephan et al., 2009); ψ is the digamma function $\psi (\alpha_{k})=\frac{\delta log\Gamma (\alpha_{k})}{\delta \alpha_{k}}$; β_k_ is the expected number of subjects whose data are believed to be generated by model *k* (so-called “data counts”); and the last line, α = α_0_ + β essentially obtains the parameters of the Dirichlet distribution by starting with the Dirichlet prior α_0_ and adding on “data counts” *β* (Stephan et al., 2009).

Once the Dirichlet parameters have been optimized as per the algorithm above, this can be used for model comparisons at the group level. One way of comparing models is to simply compare the parameter estimates, α. Another way is to calculate the multinomial parameters, 〈*r_k_*〉, that encode the posterior probability of model *k* being selected for a randomly chosen subject in the group:

(12) $$\langle r_{k}\rangle =\alpha_{k}/(\alpha_{1}+\ldots +\alpha_{k})$$

where *r_k_* is the probability of the model; the numerator of the fraction, *α_k_*, is the “occurrence” of model *k*; and the denominator (α_1_ + … + α_k_) is the sum of all model “occurrences.” This was how the PPMs were generated in the third script (“BMS3_PPMs.m”) below.

Another option for comparing models after the optimal Dirichlet parameters have been found, is to calculate the exceedance probability for a given model, as follows (Rosa et al., 2010):

(13) $$\varphi_{k}=p\left(\prod_{j\neq k}r_{k}>r_{j}|Y;\alpha \right)$$

where φ_k_ is the exceedance probability for model *k*, that is, the probability that it is more likely than any of the other models considered; r_k_ is the probability of model *k*; r_j_ is the probability of all other models considered; *Y* represents the data from all subjects and α represents the Dirichlet parameters.

Having introduced this RFX approach to VB, Stephan et al. (2009) then used both simulated and empirical data to demonstrate that when groups are heterogeneous, fixed effects analyses fail to remain sufficiently robust. Crucially, they also showed that RFX is robust to outliers, which can confound inference under FFX assumptions, when those assumptions are violated. Stephan et al. thus concluded that although RFX is more conservative than FFX, it is still the best method for selecting among competing neurocomputational models.

## Materials and Methods

### Experimental Design

**FIGURE 1:**
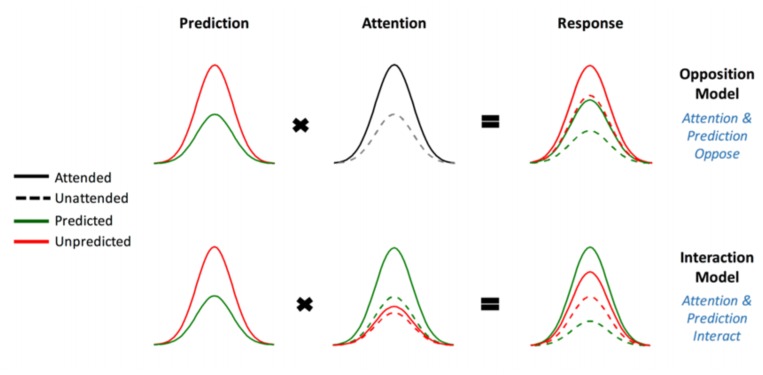
The two competing models that were evaluated using BMS. Reprinted with permission from Garrido et al. (2018) DOI: 10.1093/cercor/bhx087. Figure Published by Oxford University Press. All rights reserved. Available online at: https://academic.oup.com/cercor/advance-article/doi/10.1093/cercor/bhx087/3571164?searchresult=1. This figure is not covered by the Open-Access license of this publication. For permissions contact Journals.permissions@OUP.com.

This experiment is a direct replication of that performed by Garrido et al. (2018), apart from the omission of a “divided attention” condition. As they describe in greater detail in their paper, Garrido et al. (2018) utilized a novel audio-spatial attention task during which attention and prediction were orthogonally manipulated; this was done to evaluate the effect of surprise and attention in auditory processing (Garrido et al., 2018). The authors compared two models (shown in **Figure 1**) which may explain the effect attention has on the neural responses elicited by predicted and unpredicted events.

The original study supported the model in which attention boosts neural responses to both predicted and unpredicted stimuli, called the Opposition Model (Garrido et al., 2018). Prediction attenuates neural activity, while attention enhances this activity. Since these effects occur in opposite directions or have opposing effects, the researchers named the model (describing these effects) the Opposition Model. According to this model, attention improves the accuracy of predictions by precision weighting prediction errors more heavily. Thus, in light of this model, attention and prediction work together (in opposite directions) to improve our ability to make more accurate representations of the sensorium.

Our current study attempted to replicate the above-mentioned study with an independent dataset and employing the Bayesian methods that resembled the original study as closely as possible. The only difference was that the divided-attention condition was not administered because it was not required for the implementation and description of the BMS steps. It is hoped that the detailed description of our methods, adapted from those originally developed for fMRI by Rosa et al. (2010), prove to be useful for other EEG and/or MEG researchers. Furthermore, a replication study such as this one has the additional benefit of being responsive to the persisting replication crisis that continues to pose a significant problem for neuroscience and psychology (Hartshorne, 2012; Larson and Carbine, 2017; Szucs et al., 2017).

To this end we employed BMS to adjudicate between two competing hypotheses (see **Figure 1**), namely:

(1) Attention increases (boosts) neural responses to both predicted and unpredicted stimuli. This is formalized in the Methods section and is then called Model One – the Opposition Model.
(2) Attention boosts neural responses to predicted stimuli more than it boosts responses to unpredicted stimuli. This causes predicted attended stimuli to generate the highest neural responses, followed by attended unpredicted stimuli. This is formalized in the Methods section and is then called Model Two – the Interaction Model.

### Participants

Twenty-one healthy adults (aged between 19–64 years, M = 25.00 years, *SD* = 9.83, nine females) were recruited via the University of Queensland’s Psychology Research Participation Scheme (SONA). Exclusion criteria included any history of mental or neurological disease, any previous head injury resulting in unconsciousness, or an age outside the prescribed range (18–65 years). All participants gave both written and verbal informed consent to both the study and to having their de-identified data made available in publicly distributed databases. Participants completed practice blocks of stimulus presentation prior to undergoing the EEG recording, in order to enable them to withdraw if they found the task unpleasant or excessively challenging. (No participants wished to withdraw.) Participants were monetarily compensated for their time. This study was approved by the University of Queensland Human Research Ethics Committee.

### Task Description

Participants wore earphones with inner-ear buds (Etymotic, ER3) and were asked to follow instructions on a computer screen. Participants were asked to pay attention to the sound stream in either the left or the right ear (ignoring the sounds that were being played in the other ear). Gaussian white noise was played to both ears and an oddball sequence was played to one of the ears. During a given block, participants were tasked with listening carefully for gaps in the white noise on the side to which they had been asked to attend. They were asked to press a “1” on the numbered keyboard when they heard a single gap (lasting 90 ms) in the white noise, and a “2” when they heard a double gap (two 90 ms gaps separated by 30 ms of white noise). They were asked to ignore any tones played on both the attended and the opposite ear. This task is described in further detail, including pictorial representations, in Garrido et al. (2018).

Participants listened to eight different blocks, each 190 s in duration. Each block contained a total of 30 targets (15 single gaps and 15 double gaps, randomly distributed across the block, but never occurring within 2.5 s of each other and never occurring at the same time as a tone). Throughout each block there were also 50-ms-long pure tones being played in one of the ears, with a 450 ms inter-stimulus interval. In each block there were two tones: the standard tone (either 500 Hz or 550 Hz counterbalanced between blocks) that occurred 85% of the time, and the deviant (either 550 Hz or 500 Hz, the opposite of the standard tone and counterbalanced across blocks) that occurred 15% of the time. All sound files were created using MATLAB (**RRID**:SCR_001622; The MathWorks^3^, Inc.) with sound recordings done using Audacity^®^ (Audacity: Free Audio Editor and Recorder, **RRID**:SCR_007198) as previously described by Garrido et al. (2018). The order was counterbalanced such that no two participants received the same order of blocks.

Prior to and during the practice block/s, the volume of sound delivery was adjusted until the participant stated that they were able to hear the white noise well enough to complete the task. For each participant, an accuracy level was calculated, consisting of the percentage of white noise gaps that were correctly identified (as single or double) and responded to promptly (i.e., within 2 s of the gap/s). This was calculated separately for the practice block, which was repeated if a participant did not achieve at least 50% accuracy. Once participants achieved above 50% accuracy, they were invited to participate in the rest of the experiment. At the end of the experiment each participant’s accuracy was again calculated to ensure their accuracy level remained at least 50% (otherwise they were excluded from the study). This was to ensure that participants were attending to the task as instructed.

### EEG Data Acquisition

Using a standardized nylon head cap fitted tightly and comfortably over the scalp, 64 silver/silver chloride (Ag/AgCl) scalp electrodes were placed according to the international 10–10 system for electrode placement. As is usual for this system, electrodes were placed above and below the left eye and just lateral to the outer canthi of both left and right eyes, to generate the vertical electrooculogram (VEOG) and horizontal electrooculogram (HEOG) recordings, respectively. Continuous EEG data were recorded using a Biosemi Active Two system at a sampling rate of 1024 Hz. The onset of targets, standards and deviants were recorded with unique trigger codes at the time of delivery to the participant. Within each block, the target triggers were used for accuracy calculations, while the standard and deviant triggers were kept as the time points around which to epoch the data at a later stage.

### EEG Preprocessing

Following the collection of the raw EEG data, preprocessing was completed using SPM software (SPM12, RRID:SCR_007037; Wellcome Trust Center for Neuroimaging, London^4^). EEG data preprocessing included referencing data to the common average of all electrodes; downsampling to 200 Hz; bandpass filtering (between 0.5 to 40 Hz); eyeblink correction to remove trials marked with eyeblink artifacts (measured with the VEOG and HEOG channels); epoching using a peri-stimulus window of -100 to 400 ms and artifact rejection (with 100 uV cut-off).

#### Source Reconstruction

For source BMS, SPM12 software was used to obtain source estimates on the cortex by reconstructing the scalp activity using a single-shell head model. The forward model was then inverted with multiple sparse priors (MSP) assumptions for the variance components (Friston K. et al., 2008) under group constraints (Litvak and Friston, 2008). The entire time window of 0 to 400 ms was used to infer the most likely cortical regions that generated the data observed during this time. Images for each participant and each condition were obtained from the source reconstructions and were smoothed at full width at half maximum (FWHM) 12 × 12 × 12 mm. This source reconstruction step is available as an online script (named “BMS1_Source_ImCreate.m” and available online^5^).

### Bayesian Model Selection Maps: Implementation for M/EEG

For all data analysis steps (**Table 1**), we used SPM12 software package for MATLAB. We wrote in-house MATLAB scripts, integrated with SPM12 and now available online^6^ Copies of the scripts are also given in the **Supplementary Material**. The online scripts are divided into three BMS scripts. In the first script (BMS1_ST_ImCreate.m for spatiotemporal BMS and BMS1_Source_ImCreate.m for source BMS), we call the preprocessed EEG data and then create images for every trial, for every condition, and for every participant. The second script (BMS2_ModelSpec_VB.m) specifies the hypotheses and implements VB (as described in the Theory section). The last script (BMS3_PPMs.m) then creates PPMs.

**Table 1:** Step-by-step summary of method.

Task:	Suggested steps:
Saving the correct spm_spm_vb.m files	1. Find and open the SPM12 folder on your computer. 2. Find the spm_spm_vb.m script in that folder, and rename this to spm_spm_vb_fMRI.m. Then add the spm_spm_vb_ST.m and spm_spm_vb_source.m scripts (saved in the associated Github repository) to your SPM12 folder. 3. Before undertaking either the spatiotemporal BMS or source BMS steps, rename the currently relevant script from the above step to spm_spm_vb.m. Once you have finished the BMS steps, rename the script back to its original name, to re-identify it as being for either the spatiotemporal (‘spm_spm_vb_ST.m’) or source BMS (‘spm_spm_vb_source.m’). In this way, you will keep track of which spm_spm_vb.m script to use for whichever BMS steps you are about to do.
Creating spatiotemporal (“scalp”) PPMs:	1. BMS script 1: Change the file paths to reflect the location of ERP data. 2. Run BMS script 1: BMS1_ST_ImCreate.m. 3. Ensure the correct spm_spm_vb.m file is saved in SPM12 folder. 4. Run BMS script 2: BMS2_ModelSpec_VB.m. 5. Run BMS script 3: BMS3_PPMs.m. Threshold is set to 0.75 and adjustable.
Creating source PPMs:	1. BMS script 1: Change the file paths to reflect location of source reconstructed images. 2. Run BMS script 1: BMS1_Source_ImCreate.m. 3. Ensure the correct spm_spm_vb.m file is saved in SPM12 folder. 4. Run BMS script 2: BMS2_ModelSpec_VB.m. 5. Replace NaNs with zeros in the LogEv.nii files: BMS2b_Source_NaNtoZeros.m. 6. Run BMS script 3: BMS3_PPMs.m. Adjust probability threshold as desired.

In the model specification and VB script (BMS2_ModelSpec_VB.m), we changed individual participants’ data file structures in order to match the format that SPM typically requires to read fMRI data. This is done by first loading the relevant file path and then changing the file structure. Once these newly structured files had been saved, we next specified the models to be compared: this was done by assigning covariate weights to describe both models (please see the instructions contained within BMS2_ModelSpec_VB.m on Github). The Opposition Model was assigned weights of [1, 2, 2, and 3] for the unattended predicted, attended predicted and unattended unpredicted, and attended unpredicted, respectively. The Interaction Model was assigned weights of [1, 4, 2, and 3] for the same conditions.

These covariate weights essentially describe the assumed relationship between the different conditions according to a given model. For example, using [1, 2, 2, and 3] as employed in the Opposition Model, means that according to the Opposition Model, the unattended predicted condition (the first condition with an assigned weight of 1) evokes the smallest activity, whereas the attended unpredicted (the fourth condition with a weight of 3) has the greater activity, and both attended predicted and unattended unpredicted (second and third conditions with an equal weight of 2) are in between the former two conditions and indistinguishable in magnitude from each other.

**FIGURE 2:**
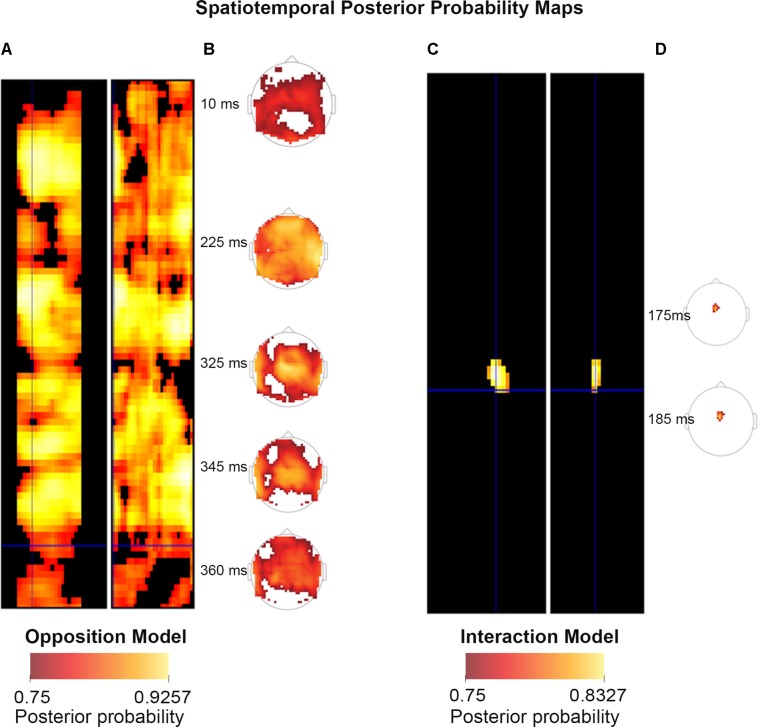
Scalp Posterior Probability Maps of the two competing models over space and time. (The scalp images include the participant’s nose, pointing upward, and ears, visible as if viewed from above.) These maps display all posterior probabilities exceeding 75% over space and time for both models. The left sides of both panels **(A,C)** both depict the temporal information, showing the model probabilities at each point in time from 0 ms (when the tone was played, at the top of the diagrams) to 400 ms after the stimulus presentation (at the bottom of the diagram), across the surface of the scalp (which traverses the width of the panels). The right sides **(B,D)** show the spatial locations of the probability clusters which exceeded the threshold of 75% probability. Panels **(B)** and **(D)** were generated using the spatiotemporal visualization tools developed by Jeremy Taylor. These tools are available at: https://github.com/JeremyATaylor/Porthole.

We then created log-evidence images, representing the log-model evidences, for both models (separately), for every participant (individually) at every voxel. In the case of spatiotemporal (scalp-level) BMS, each voxel was representative of a specific spatiotemporal location within the peristimulus time window (0 to 400 ms) and topologically across the scalp, such that the first two dimensions of the voxel refer to the space across the scalp and the third dimension is time (as shown in **Figure 2**). Conversely, in the source BMS (which began with the source reconstruction steps described above), each voxel was representative of an inferred location in three-dimensional source space. Once log-evidence images had been created, these were smoothed with a 1 mm half-width Gaussian kernel.

In summary, one can create PPMs or log evidence maps in sensor or source space. In sensor space, this involves creating a two-dimensional image over the scalp surface and equipping the space with a peristimulus time dimension. This creates PPMs over the scalp surface and peristimulus time, enabling one to identify regionally and temporally specific effects due to a particular model, relative to other contrasts. Alternatively, one can create three-dimensional PPMs in source space, following source reconstruction.

The core SPM script that allows VB to be used on fMRI data is named spm_spm_vb.m and is found in the SPM12 package, downloadable from the SPM site. This core script was edited in order to adapt the VB method for EEG, as follows. Changes were made such that different data structures could be read in the same way that fMRI data would usually be read. Furthermore, high-pass filtering steps were removed as these only apply to low-frequency drifts associated with fMRI data. The specific changes made between the original script and the altered one to be used for spatiotemporal BMS areaccessible online (goo.gl/ZVhPT7). For the source BMS steps, the same changes were left in place as outlined above, and in addition, the required minimum cluster size was changed from 16 to 0 voxels to allow for visualization of all clusters of any size. The specific differences between the original and source BMS versions of the spm_spm_vb script are accessible online (goo.gl/WXAo67).

**FIGURE 3:**
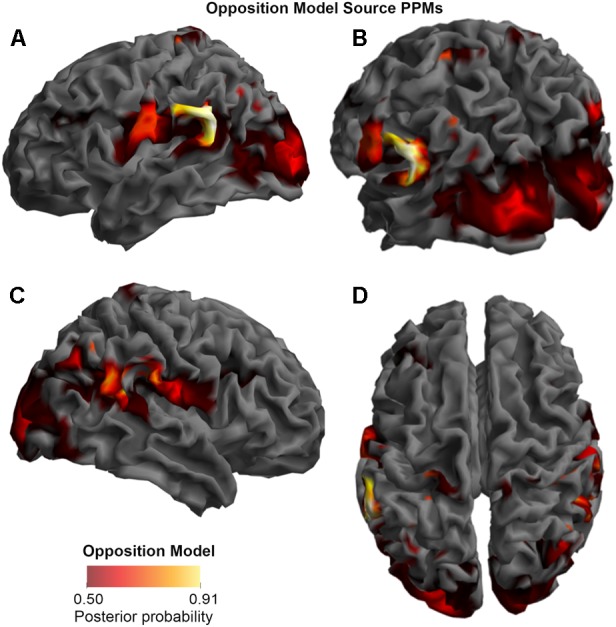
Source Posterior Probability Map for the Opposition Model (that is, reconstructed images representing the model inference at the group level for this model), thresholded at > 50% posterior probability. **(A)** View from the left side. **(B)** View from the left side, from the posterior (back) end. **(C)** View from the right side. **(D)** View from above.

**FIGURE 4:**
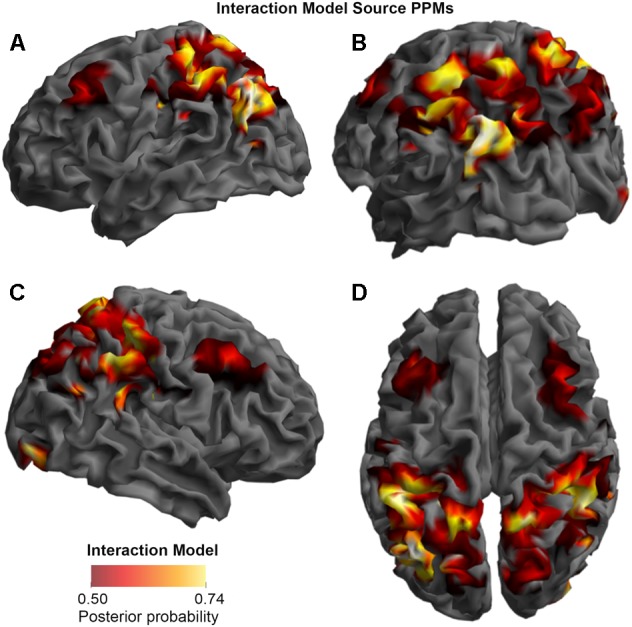
Source Posterior Probability Map for the Interaction Model (that is, reconstructed images representing the model inference at the group level for this model), thresholded at > 50% posterior probability. **(A)** View from the left side. **(B)** View from the left side, from the posterior (back) end. **(C)** View from the right side. **(D)** View from above.

In the final step (BMS3_PPMs.m), the SPM Batch Editor was used to apply a RFX approach to the group model evidence data in a voxel-wise manner, thus translating the log-evidence images from the previous step into PPMs (similar to how Rosa et al. (2010) have produced PPMs previously for fMRI data). The maps, displayed in the **Figures 2–4**, were generated by selecting threshold probabilities of 75% for the spatiotemporal maps (**Figure 2**) and 50% for the source maps (**Figures 3** and **4**). This threshold can be adjusted by the user. EPMs can also be displayed by selecting the relevant setting in the final script (please see the instructions on Github).

## Results

The raw dataset for this study can be found; on Figshare (EEG_Auditory_Oddball_Raw_Data repository^7^; Harris et al., 2018a).

The preprocessed dataset for this study can also be found on Figshare (EEG_Auditory_Oddball_Preprocessed_Data repository^8^; Harris et al., 2018b).

### Scalp – Spatiotemporal

**Figure 2** shows scalp (spatiotemporal) PPMs of the two competing models over space and time. These maps display all posterior probabilities exceeding 75% over space and time for both models. As can be seen in the figure, spatiotemporal BMS results revealed that Model One (the Opposition Model) was by and large the superior model. The Opposition Model had model probabilities exceeding 75% across the majority of later time points (with most significant clusters between 225–360 ms), and over most frontocentral and bilateral channel locations, as shown in (**A**). On the other hand, as shown in (**C**), the Interaction Model did have over 75% model probability centrally between 175–185 ms, which is within the mismatch negativity (MMN) time window. These findings replicate those of Garrido et al. (2018), and strongly support the implications discussed in great depth in that paper.

### Source

**FIGURE 5:**
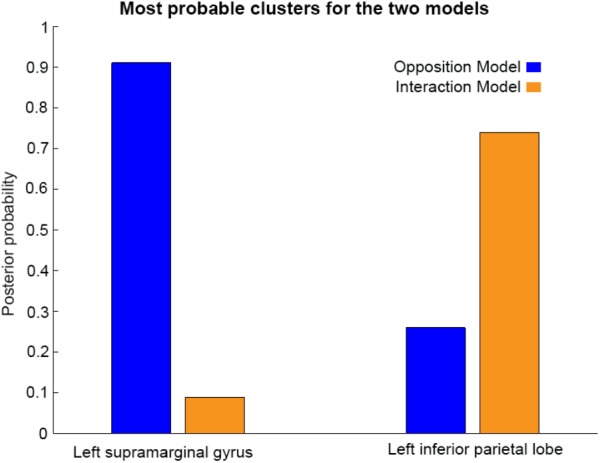
Comparison of the posterior probabilities for the two models at the location of the highest-probability cluster of the Opposition Model (left) and the location of the highest-probability cluster of the Interaction Model (right). The left supramarginal gyrus cluster, which was the highest probability cluster for the Opposition Model (left), was located at Montreal Neurological Institute (MNI) coordinates (62, –42, 30), while the left inferior parietal lobe cluster, which was the highest probability cluster for the Interaction Model, was located at MNI coordinates (–54, –32, 46).

As shown in **Figures 3**, **4**, and **5**, source BMS results also favored the Opposition Model, with higher model probability over the left supramarginal gyrus (with 91% model probability over a relatively large cluster, *K*_E_ = 6115), the right superior temporal gyrus (with 87% model probability over a cluster with *K*_E_ = 5749) as well as over parts of the left inferior parietal lobe, right inferior parietal lobe and left postcentral gyrus. Having said this, the Interaction Model also had two large clusters, albeit with lower model probabilities compared to the Opposition Model’s highest-probability clusters: specifically, the Interaction Model had a cluster of size *K*_E_ = 6346 over the left inferior parietal lobe and a cluster of size *K*_E_ = 5353 over the right inferior parietal lobe (with 74% model probability in both places).

**Figures 3** and **4** show that different brain regions are likely to perform different computations best described by the Opposition and Interaction Models, respectively. Furthermore, **Figure 5** compares the magnitude of the calculated posterior probabilities, at the locations of the highest probability cluster for both models. The possible functional reasons for the different anatomical locations that emerge for the two different models may be an interesting subject for future study, but fall outside the scope of this methods paper. In any case, the usefulness of this probability mapping approach illustrated in **Figures 2**, **3**, and **4**, lies in the ability to pinpoint where and when given computations are likely to be performed in the brain.

## Discussion

This paper shows how to use RFX BMS mapping methods for M/EEG data analysis. This method was originally developed for fMRI by Rosa et al. (2010), and provides a way of displaying the probabilities of different cognitive models at different timepoints and brain locations, given a neuroimaging dataset. We aimed to provide an in-depth explanation, written in a didactical manner, of the BMS and posterior probability mapping steps that were successfully used by Garrido et al. (2018) in their recent EEG paper.

Being a Bayesian approach to hypothesis-testing, the method described here provides multiple advantages over frequentist inference methods. The first of these advantages is that VB allows for comparisons between non-nested models. Consequently, it is especially useful in the context of model-based neuroimaging (Montague et al., 2004; O’Doherty et al., 2007; Rosa et al., 2010; Garrido et al., 2018). Another advantage is that the probability of the null hypothesis itself can be assessed (instead of simply being, or failing to be, rejected). A final advantage is that, although only two models were compared here, the same method can also be applied to any arbitrary number of models. For example, the analyses described here could proceed slightly differently, based on the same data but introducing another (or multiple other) model/s against which to compare the Opposition and Interaction Models. Potentially, any number of theoretically motivated models could be considered. Considering all of these advantages, the method described here should prove useful in a wide variety of M/EEG experiments.

In summary, we have shown here how to adapt BMS maps, originally developed for fMRI data by Rosa et al. (2010), to M/EEG data analysis. It is hoped that the reporting of analytical methods such as these, as well as the availability of all the code and dataset, will not only contribute to the Open Science movement, but may also encourage other researchers to adopt this novel M/EEG data analysis method in a way that is useful for addressing their own neuroscience questions. We postulate that the use of this Bayesian model mapping of M/EEG data to adjudicate between competing computational models in the brain, both at the scalp and source level, will be a significant advancement in the field of M/EEG neuroimaging and may provide new insights in cognitive neuroscience.

## Author Contributions

MG designed the study and the analysis methods. ER wrote the code and adapted SPM scripts from fMRI to M/EEG. CH and RR collected and analyzed the data, and organized the data and code for sharing. CH wrote the first draft of the manuscript. ER, RR, and MG edited the manuscript.

## Conflict of Interest Statement

The authors declare that the research was conducted in the absence of any commercial or financial relationships that could be construed as a potential conflict of interest.

## Footnotes

1 https://figshare.com/s/1ef6dd4bbdd4059e3891

2 https://github.com/ClareDiane/BMS4EEG

3 http://www.mathworks.com

4 http://www.fil.ion.ucl.ac.uk/spm/

5 https://github.com/ClareDiane/BMS4EEG

6 https://github.com/ClareDiane/BMS4EEG

7 https://figshare.com/s/1ef6dd4bbdd4059e3891

8 https://figshare.com/s/c6e1f9120763c43e6031
