# Supplementary material for: Bayesian Model Selection Maps for Group Studies Using M/EEG Data
Source: Front Neurosci. 2018 Sep 28;12:598. doi: 10.3389/fnins.2018.00598 (PMC6190865; doi:10.3389/fnins.2018.00598)
Supplement: Supplementary file 1 [file Presentation_1.PDF]

Supplementary Material

Bayesian Model Selection Maps for group studies using M/EEG data

Clare D. Harris†*, Elise G. Rowe†, Roshini Randeniya*, Marta I. Garrido

† Equal first authorship

*** Correspondence:** Corresponding Author: clare.harris@uqconnect.edu.au

1 Supplementary Data

The raw dataset for this study can be found on Figshare (EEG_Auditory_Oddball_Raw_Data depository, https://figshare.com/s/1ef6dd4bbdd4059e3891).

Following the collection of the raw EEG data, preprocessing was completed using Statistical Parametric Mapping (SPM) software (SPM12, RRID:SCR_007037; Wellcome Trust Centre for Neuroimaging, London: http://www.fil.ion.ucl.ac.uk/spm/). EEG data preprocessing included referencing data to the common average of all electrodes; downsampling to 200 Hz; bandpass filtering (between 0.5 to 40 Hz); eyeblink correction to remove trials marked with eyeblink artefacts (measured with the VEOG and HEOG channels); epoching using a peri-stimulus window of -100 to 400 ms; artefact rejection (with 100 uV cut-off); low-pass filtering (40 Hz; to remove any high frequency noise) and baseline correction (-100 to 0 ms window). The preprocessed dataset is stored on Figshare (EEG_Auditory_Oddball_Preprocessed_Data depository, https://figshare.com/s/c6e1f9120763c43e6031).

2 Supplementary Code

For all data analysis steps, we used SPM12 software package for MATLAB. ER wrote in-house MATLAB scripts, integrated with SPM12 and available online on Github, where you can track any future updates to the code (https://github.com/ClareDiane/BMS4EEG).

To use these scripts, you will first need the following:

- EEG data pre-processed in SPM prior to averaging across conditions (you may use your own data or the pre-processed data provided at the link given above, which is downloadable and ready to use)
- Statistical Parametric Mapping (SPM12) toolbox installed
- MATLAB: version 2016b advised (because this is the system for which the scripts are optimised)
- Advised: Windows computer and operating system (this is the system for which the scripts are optimised)

The online scripts are divided into three BMS scripts. In the first script (BMS1_ST_ImCreate.m), we call the preprocessed ERP data and then create images for every trial, for every condition, and for every participant. The second script (BMS2_ModelSpec_VB.m) specifies the computational model

and implements Variational Bayes (as described in the Theory section of the accompanying article). The last script (BMS3_PPMs.m) then creates Posterior Probability Maps.

For saving the correct spm_spm_vb.m files, the suggested steps are:

1. Find and open the spm12 folder on your computer.
2. Find the spm_spm_vb.m script in that folder, and rename this to spm_spm_vb_fMRI.m. Then add the spm_spm_vb_ST.m and spm_spm_vb_source.m scripts (which you can obtain from https://github.com/ClareDiane/BMS4EEG) to your SPM12 folder.
3. Before undertaking either the spatiotemporal BMS or source BMS steps, rename the currently-relevant script from the above step to spm_spm_vb.m. Once you have finished the BMS steps, rename the script back to its original name, to re-identify it as being for either the spatiotemporal ('spm_spm_vb_ST.m') or source BMS ('spm_spm_vb_source.m'). In this way, you will keep track of which spm_spm_vb.m script to use for whichever BMS steps you are about to do.

For spatiotemporal ("scalp") PPMs:

1. BMS script 1: Change the file paths to reflect the location of ERP data
2. Run BMS script 1: BMS1_ST_ImCreate.m
3. Ensure the correct spm_spm_vb.m file is saved in SPM12 folder
4. Run BMS script 2: BMS2_ModelSpec_VB.m
5. Run BMS script 3: BMS3_PPMs.m. Threshold set to 0.75 and adjustable..

For source PPMs:

1. BMS script 1: Change the file paths to reflect location of source reconstructed images
2. Run BMS script 1: BMS1_Source_ImCreate.m.
3. Ensure the correct spm_spm_vb.m file is saved in SPM12 folder
4. Run BMS script 2: BMS2_ModelSpec_VB.m
5. Replace NaNs with zeros in the LogEv.nii files: BMS2b_Source_NaNtoZeros.m
6. Run BMS script 3: BMS3_PPMs.m. Adjust probability threshold as desired.

A copy of the scripts is provided below; for the live copies, including any potential future updates, please see the scripts stored on Github (https://github.com/ClareDiane/BMS4EEG).

BMS1_ST_ImCreate.m

```
%% BMS 1: Spatiotemporal Image Creation Script

% This script creates trial images for every trial, for every participant

% and for every condition (in the same way that one would create images

% of each condition's average per participant in flexible full factorial

% designs, except that  this script uses the file prior to averaging across

% conditions. (For example, use the 'afdespm12.mat' file per participant

% instead of the final 'bfmaeafdespm12.mat' file. The full names of the

% preprocessed files we used are aeTBafdMpspmeeg_[sub-no]_EEG.mat.)


% N.B. No smoothing is performed in this step (apply if required).


% Requirements for this script:

% - Statistical Parametric Mapping (SPM12) toolbox (SPM12) installation

% - EEG data preprocessed in SPM prior to averaging across conditions


% This script was run on EEG data that were preprocessed in the following

% order (but can also be run on EEG data preprocessed using SPM in

% any chosen order of steps - selected files must be PRIOR to averaging):

%   1) conversion of data files to MATLAB files

%   2) montaging by referencing all electrodes against each other

%   3) downsampling to 200 Hz;

%   4) bandpass filtering (between 0.5 to 40 Hz)

%   5) eyeblink correction to remove trials marked with eyeblink artefacts
```

```
%     (measured with the VEOG and HEOG channels)
%   6) epoching using a peri-stimulus window of -100 to 400 ms
%   7) artefact rejection (with 100 uV cut-off)
%   8) robust averaging
%   9) low-pass filtering (40 Hz; to remove any high frequency noise)
%   10) baseline correction (-100 to 0 ms window).
% Note: pre-processed data files we used were from after step 7 was applied

% We used data from an auditory oddball paradigm (as described in
% Garrido et al., 2017), obtained from the Queensland Brain Institute,
% Australia, using a 64 channel EEG Biosemi system. For the raw data,
% please see: https://figshare.com/s/1ef6dd4bbdd4059e3891 and for the
% preprocessed data, please see: https://figshare.com/s/c6e1f9120763c43e6031

% For further information on data collection and on this analysis method
% see: Garrido, M.I., Rowe, E.G., Halasz, V., & Mattingley, J. (2017).
% Bayesian mapping reveals that attention boosts neural responses to
% predicted and unpredicted stimuli. Cerebral Cortex, 1-12.
% DOI: 10.1093/cercor/bhx087

% If you use these scripts, please cite:
% Harris C.D., Rowe, E.G., Randeniya, R. and Garrido, M.I. (2018).
% Bayesian Model Selection Maps for group studies using M/EEG data.

% Scripts written by Elise Rowe, July 2016.
```

```
% Further changes made by Clare Harris and Elise Rowe, April 2017.

% Please note that these scripts have been optimised for MATLAB 2016b.

%%%%%%%%%%%%%%%%%%%%%%%%%%%%%%%%%%%%%%%%%%%%%%%%%%%%%%%%%%%%%%%%%%%%%%%%%%%%%%%%%%%%%%%%%%%%%%%%%%%%%%%%%%%%%%%%%%%%%%%%%%%%%%%%%%%%%%%%%%%

%% Create images for each trial for each participant

clear all;

Participants = [1:20] % Enter your participant numbers in ascending order

% Ensure preprocessed images are contained in folders numbered by
% participant number -- see below for full filepath example:
% ['C:\FolderExample1\SubfolderExample1\1\aeTBafdMpspmeeg_1_example.mat'];

filePath = ['C:\FolderExample1\SubfolderExample1\'] %enter the file path

for cycle = 1:length(Participants) %loop through participant list
   PP = Participants(cycle);

   spm('defaults', 'EEG');

   spm_jobman('initcfg');
```

```
filename = [filePath num2str(PP) '\aeTBafdMpspmeeg_' num2str(PP) '_example.mat'];
% Above, enter the filename of the preprocessed images.

matlabbatch{1}.spm.meeg.images.convert2images.D = {filename};
matlabbatch{1}.spm.meeg.images.convert2images.mode = 'scalp x time'; %spatiotemporal data
matlabbatch{1}.spm.meeg.images.convert2images.conditions = {
'Attended_Standards'
'Unattended_Standards'
'Attended_Deviants'
'Unattended_Deviants'};
 % In the above lines, name your experimental conditions if using your
 % data. If using the data on Figshare, please see Garrido et al. (2017)
 % for explanations of each condition written above.

matlabbatch{1}.spm.meeg.images.convert2images.channels{1}.type = 'EEG';
matlabbatch{1}.spm.meeg.images.convert2images.timewin = [0 400];
% Our chosen desired peristimulus time interval was 0 to 400 ms

matlabbatch{1}.spm.meeg.images.convert2images.freqwin = [-Inf Inf];
% Frequency window is here set to include all frequencies

matlabbatch{1}.spm.meeg.images.convert2images.prefix = 'EEG_allTrials_forBMS_';
% Choose your desired prefix for the resulting saved files
% NB: please take note of the resulting saved file names, because you
% will need to enter these at the start of the next script.
```

```
    spm_jobman('serial',matlabbatch);

end
```

BMS1_Source_ImCreate.m

```
%% Source Level Analysis for BMS (With Group Inversion)
% This script needs to be run on the preprocessed EEG data before source BMS can proceed.
% This script batches together the data files (therefore Group Inversion will be
% done automatically). This is the prefered method for group analysis.

%Steps completed by this script include:
% 1. Source space modeling (specifying head model template : MRI in MNI coordinates)
% 2. Data co-registration (Specify locations that can link the MRI MNI
% coordinates and EEG space (fiducials))
% 3. Forward computation (Computing the effect which each dipole on the
% cortical mesh will have on the sensors)
% 4. Inverse reconstruction, and
% 5. Summarising results of inverse reconstruction as an image.

% The final output file is 'bfmaeTBafdMspmeeg_1001_[Name]_1_t0_400_f_1'
% up to 'bfmaeTBafdMspmeeg_1001_[Name]_1_t0_400_f_4'

% Requirements for this script:
% - Statistical Parametric Mapping (SPM12) toolbox (SPM12) installation
% - EEG data preprocessed in SPM prior to averaging across conditions
```

```
% This script was run on EEG data that were preprocessed in the following
% order (but can also be run on EEG data preprocessed using SPM in
% any chosen order of steps - selected files must be PRIOR to averaging):
%   1) conversion of data files to MATLAB files
%   2) montaging by referencing all electrodes against each other
%   3) downsampling to 200 Hz;
%   4) bandpass filtering (between 0.5 to 40 Hz)
%   5) eyeblink correction to remove trials marked with eyeblink artefacts
%      (measured with the VEOG and HEOG channels)
%   6) epoching using a peri-stimulus window of -100 to 400 ms
%   7) artefact rejection (with 100 uV cut-off)
%   8) robust averaging
%   9) low-pass filtering (40 Hz; to remove any high frequency noise)
%   10) baseline correction (-100 to 0 ms window).
% Note: pre-processed data files we used were from after step 7 was applied

% We used data from an auditory oddball paradigm (as described in
% Garrido et al., 2017), obtained from the Queensland Brain Institute,
% Australia, using a 64 channel EEG Biosemi system. For the raw data,
% please see: https://figshare.com/s/1ef6dd4bbdd4059e3891 and for the
% preprocessed data, please see: https://figshare.com/s/c6e1f9120763c43e6031

% For further information on data collection and on this analysis method
% see: Garrido, M.I., Rowe, E.G., Halasz, V., & Mattingley, J. (2017).
```

```
% Bayesian mapping reveals that attention boosts neural responses to
% predicted and unpredicted stimuli. Cerebral Cortex, 1-12.
% DOI: 10.1093/cercor/bhx087

% If you use these scripts, please cite:
% Harris C.D., Rowe, E.G., Randeniya, R. and Garrido, M.I. (2018).
% Bayesian Model Selection Maps for group studies using M/EEG data.

% Generated using SPM12 by Roshini Randeniya, April 2017.

% Please note that these scripts have been optimised for MATLAB 2016b.

%%%%%%%%%%%%%%%%%%%%%%%%%%%%%%%%%%%%%%%%%%%%%%%%
%%%%%%%%%%%%%%%%%%%%%%%%%%%

%% Create images for each trial for each participant
clear all

Participants = [1:20]'; % Enter range of participant numbers in ascending order

filepath = 'C:\FolderExample1\SubfolderExample1\'; % Enter the file path
filesuffix = '_[Name].mat'; % Enter the file suffix

%% Run Script
spm('defaults', 'EEG')
```

```
spm_jobman('initcfg');


%  Template, Coregister, Forward Model
for PP = 1:length(Participants)
   currname = num2str(Participants(PP));
   matlabbatch{1}.spm.meeg.source.headmodel.D{PP,1} = [filepath currname '\aeTBafdMpspmeeg_' ...
       currname filesuffix];
end


matlabbatch{1}.spm.meeg.source.headmodel.val = 1;
matlabbatch{1}.spm.meeg.source.headmodel.comment = 'Source';
matlabbatch{1}.spm.meeg.source.headmodel.meshing.meshes.template = 1;
matlabbatch{1}.spm.meeg.source.headmodel.meshing.meshres = 2; % 1 = coarse,
% 2 = normal, 3 = fine
matlabbatch{1}.spm.meeg.source.headmodel.coregistration.coregdefault = 1; % 1= yes
matlabbatch{1}.spm.meeg.source.headmodel.forward.eeg = 'EEG BEM';
matlabbatch{1}.spm.meeg.source.headmodel.forward.meg = 'Single Shell';


%Invert, Window
matlabbatch{2}.spm.meeg.source.invert.D(1) = cfg_dep('Head model specification: M/EEG dataset(s) with a forward model', ...
   substruct('.','val', '{}',{1}, '.','val', '{}',{1}, '.','val', '{}',{1}, '.','val', '{}',{1}), substruct('.','D'));
matlabbatch{2}.spm.meeg.source.invert.val = 1;
matlabbatch{2}.spm.meeg.source.invert.whatconditions.all = 1;
matlabbatch{2}.spm.meeg.source.invert.isstandard.standard = 1;
```

matlabbatch{2}.spm.meeg.source.invert.modality = {'EEG'};

%Window, Images

matlabbatch{3}.spm.meeg.source.results.D(1) = cfg_dep('Source inversion: M/EEG dataset(s) after imaging source reconstruction', ...

substruct('.','val', '{}',{2}, '.','val', '{}',{1}, '.','val', '{}',{1}, '.','val', '{}',{1}), substruct('.','D'));

matlabbatch{3}.spm.meeg.source.results.val = 1;

matlabbatch{3}.spm.meeg.source.results.woi = [0 400]; % time of interest

matlabbatch{3}.spm.meeg.source.results.foi = [0 0]; % frequency window specify

matlabbatch{3}.spm.meeg.source.results.ctype = 'trials'; % 'evoked' 'induced' or single 'trials'

matlabbatch{3}.spm.meeg.source.results.space = 1; % 1 = MNI or Native

matlabbatch{3}.spm.meeg.source.results.format = 'image';

matlabbatch{3}.spm.meeg.source.results.smoothing = 12; % mm voxel smoothing value (the default is 8x8x8 mm

%however the setting for this script is 12x12x12 mm)

spm_jobman('serial',matlabbatch);

clear all

BMS2_ModelSpec_VB.m

%% BMS 2: Model Specification and Log-Evidence Estimation via Variational Bayes

% This script takes the images from the previous steps (both for

% spatiotemporal data images or source reconstruction images), and allows

% the user to introduce covariate weights to define the models to be tested.

% These models will be compared via Bayesian Model Selection (BMS) using
% the BMS 3 scripts.

% Once the models are defined, this script calls the modified spm_spm_vb.m
% script to implement Variational Bayes (VB) to calculate the log of the
% model evidence (LogEv for short) for each of the defined models,
% for each participant in the M/EEG dataset. The results are stored in
% files ('LogEv.nii' image files) which are kept in folders for each
% participant. At the end of this script you will have (n*participants)
% unique LogEv.nii files, where n is the number of models you defined at
% the start of the script.

% Model specification and estimation is run for every participant individually.
% These LogEv files will be used in the final BMS step, in the next script.

% Please take note of the additional steps that are required before
% performing this script (these steps are listed below).

% For both spatiotemporal and source BMS steps, you need to save the
% relevant spm_spm_vb.m script in the SPM12 folder on your computer.
% Please see this Github repository for the correct script for the
% spatiotemporal and for the source BMS steps.

%For spatiotemporal BMS steps: run this script after the BMS Image Creation
% Script is complete for all participants

%For source BMS steps: run this script after the BMS Image Creation and

% Group Source Inversion have both been completed for all participants

% Requirements for this script:

% - SPM12 installation

% - Trial images per participant and condition saved from completed

%   BMS1_ST_ImCreat.m Script for spatiotemporal BMS, or from the completed

%   BMS1_source_ImCreate.m' script for source level analysis

% - the correct spm_spm_vb.m script saved in the SPM12 folder

% either for source or spatiotemporal EEG analyses (see the corresponding

% instructions in this GitHub respository)

% We used data from an auditory oddball paradigm (as described in

% Garrido et al., 2017), obtained from the Queensland Brain Institute,

% Australia, using a 64 channel EEG Biosemi system. For the raw data,

% please see: https://figshare.com/s/1ef6dd4bbdd4059e3891 and for the

% preprocessed data, please see: https://figshare.com/s/c6e1f9120763c43e6031

% For further information on data collection and on this analysis method

% see: Garrido, M., Rowe, E., Halasz, V., & Mattingley, J. (2017).

% Bayesian mapping reveals that attention boosts neural responses to

% predicted and unpredicted stimuli. Cerebral Cortex, 1-12.

% DOI: 10.1093/cercor/bhx087

% If you use these scripts, please cite:

```
% Harris C.D., Rowe, E.G., Randeniya, R. and Garrido, M.I. (2018).
% Bayesian Model Selection Maps for group studies using M/EEG data.

% Scripts written by Elise Rowe, July 2016.
% Further changes made by Clare Harris and Elise Rowe, April 2017.

% Please note that these scripts have been optimised for MATLAB 2016b.

%Potential time points where the user may need to make manual selection:
% 1) When the script first runs, if the directory chosen is not the same
%   folder as the one where the user currently is, then the user will be
%   asked if they wish to change folders and will then need to hit "Enter" or
%   click "Yes" for the script to run.
% 2) There may be two dialogue boxes warning of a file being "overwritten",
%   which you may respond to in the affirmative, however in standard Windows
%   laptops the script may still run even if the user does not respond to
%   these dialogue boxes.

%%%%%%%%%%%%%%%%%%%%%%%%%%%%%%%%%%%%%%%%%%%%%%%%%%%%%%%%%%%%%%%%%%%%%%%%%%

%% Start BMS 2 Process
clear all

Participants = [1:20]; % Enter range of participant numbers in ascending order
```

```
fileNames = {'condition_Attended_Standards', ...
    'condition_Unattended_Standards','condition_Attended_Deviants', ...
    'condition_Unattended_Deviants',}
% Enter your experimental conditions above. For an explanation of
% the conditions listed above, please see Garrido et al. (2017).

%% Collect all image names from an individual participant
for cycle = 1:length(Participants)
    PP = Participants(cycle)

    cd(['C:\FolderExample1\SubfolderExample1\' num2str(PP) ...
        '\EEG_allTrials_forBMS_aeTBafdMpspmeeg_' num2str(PP) '_example'])
    % Enter the name and location of the folders for each participant.
    % Check that the folder you are referring to above is the same as the
    % folder that you created in the previous step.

    for nextCycle = 1:length(fileNames)
        useTheseIm = cell2mat(fileNames(nextCycle));

        thisPart = ['aeafdMpspmeeg_P' num2str(PP)];

        %List names of all images in folder for each trial name
        imagefiles = dir([useTheseIm '.nii']);
        filenames = {imagefiles(:).name};
        loadName = cell2mat(filenames);
```

```
        %Collect all names of images in the folder for this trial name
        metaFileNames = spm_vol([loadName])
        metaInput = struct2cell(metaFileNames)
        inputFileNames = metaInput(1,:)

        inputFileNamesBMS = inputFileNames'

        for addOn = 1:length(inputFileNamesBMS)
            appendFileName = cell2mat(inputFileNamesBMS(addOn,:));
            inputFileNamesBMS{addOn,:} = [appendFileName ',' num2str(addOn)];
        end

        filename = ['P' num2str(PP) '_' num2str(useTheseIm) '_BMS_Trials.mat']

        save(filename, 'inputFileNamesBMS')
        clear inputFileNamesBMS
    end
end

%% Model Specification (insert Regressor Covariates for Models)

for cycle = 1:length(Participants)
    PP = Participants(cycle);
    for model = 1:2 % Change this to reflect the number of models considered
```

```
cd(['C:\FolderExample1\SubfolderExample1\' num2str(PP) ...
    '\EEG_allTrials_forBMS_aeTBafdMpspmeeg_' num2str(PP) '_example'])

load(['P' num2str(PP) '_' cell2mat(fileNames(1)) '_BMS_Trials.mat'])
scan1 = inputFileNamesBMS;
load(['P' num2str(PP) '_' cell2mat(fileNames(2)) '_BMS_Trials.mat'])
scan2 = inputFileNamesBMS;
load(['P' num2str(PP) '_' cell2mat(fileNames(3)) '_BMS_Trials.mat'])
scan3 = inputFileNamesBMS;
load(['P' num2str(PP) '_' cell2mat(fileNames(4)) '_BMS_Trials.mat'])
scan4 = inputFileNamesBMS;

spm('defaults', 'EEG');
spm_jobman('initcfg');

if model == 1
    makeFolder = ['C:\FolderExample1\SubfolderExample1\P' num2str(PP) '\BMS_Model1'];
    mkdir(makeFolder)
    designDir = makeFolder;
else
    makeFolder = ['C:\FolderExample1\SubfolderExample1\P' num2str(PP) '\BMS_Model2'];
    mkdir(makeFolder)
    designDir = makeFolder;
end

%% Model definition/specification step
```

```
% Define your model weights (higher values = larger responses)
modelOne_Weights = [2,1,3,2]; % apply covariate weights according to first model
modelTwo_Weights = [4,1,3,2]; % apply covariate weights for model two

matlabbatch{1}.spm.stats.factorial_design.dir = {designDir};

%%
matlabbatch{1}.spm.stats.factorial_design.des.anova.icell(1).scans = (scan1)
matlabbatch{1}.spm.stats.factorial_design.des.anova.icell(2).scans = (scan2)
matlabbatch{1}.spm.stats.factorial_design.des.anova.icell(3).scans = (scan3)
matlabbatch{1}.spm.stats.factorial_design.des.anova.icell(4).scans = (scan4)

%%
%%
matlabbatch{1}.spm.stats.factorial_design.des.anova.dept = 0;
matlabbatch{1}.spm.stats.factorial_design.des.anova.variance = 1;
matlabbatch{1}.spm.stats.factorial_design.des.anova.gmsca = 0;
matlabbatch{1}.spm.stats.factorial_design.des.anova.ancova = 0;

%% Input the Covariates
%(multiplies the covariate weights per condition trial number)
if model == 1
   matlabbatch{1}.spm.stats.factorial_design.cov.c = ...
      [(ones(1,length(scan1)))*(modelOne_Weights(1)) ...
      (ones(1,length(scan2)))*(modelOne_Weights(2)) ...
```

```
        (ones(1,length(scan3)))*(modelOne_Weights(3)) ...
        (ones(1,length(scan4)))*(modelOne_Weights(4))]';
    matlabbatch{1}.spm.stats.factorial_design.cov.cname = 'Model1';
    matlabbatch{1}.spm.stats.factorial_design.cov.iCFI = 1;
    matlabbatch{1}.spm.stats.factorial_design.cov.iCC = 5;
elseif model == 2
    matlabbatch{1}.spm.stats.factorial_design.cov.c = ...
        [(ones(1,length(scan1)))*(modelTwo_Weights(1)) ...
        (ones(1,length(scan2)))*(modelTwo_Weights(2)) ...
        (ones(1,length(scan3)))*(modelTwo_Weights(3)) ...
        (ones(1,length(scan4)))*(modelTwo_Weights(4))]';
    matlabbatch{1}.spm.stats.factorial_design.cov.cname = 'Model2';
    matlabbatch{1}.spm.stats.factorial_design.cov.iCFI = 1;
    matlabbatch{1}.spm.stats.factorial_design.cov.iCC = 5;
end

matlabbatch{1}.spm.stats.factorial_design.masking.tm.tm_none = 1;
matlabbatch{1}.spm.stats.factorial_design.masking.im = 1;
matlabbatch{1}.spm.stats.factorial_design.masking.em = {''};
matlabbatch{1}.spm.stats.factorial_design.globalc.g_omit = 1;
matlabbatch{1}.spm.stats.factorial_design.globalm.gmsca.gmsca_no = 1;
matlabbatch{1}.spm.stats.factorial_design.globalm.glonorm = 1;

spm_jobman('serial',matlabbatch);
clear matlabbatch; clear scan1; clear scan2; clear scan3; clear scan4;
```

```
    end
end

%% VB Model specification and LogEvidence calculation

for cycle = 1:length(Participants)
    PP = Participants(cycle);
    for model = 1:2 % Change this to reflect the number of models considered

        %Load the SPM files for each participant
        if model == 1
            filePath = ['C:\FolderExample1\SubfolderExample1\P' num2str(PP)
'\BMS_Model1\SPM.mat']
            designDirBMS = filePath;
        elseif model == 2
            filePath = ['C:\FolderExample1\SubfolderExample1\P' num2str(PP)
'\BMS_Model2\SPM.mat']
            designDirBMS = filePath;
        end

        %% Adapting to EEG data
        % The below five lines change the data structure so that the EEG data is
        % able to be examined with scripts that were originally designed for fMRI
        load(filePath)
        SPM.Sess(1).row = size(SPM.xX.X,1);
        SPM.Sess(1).col = SPM.xX.iC;
```

```
save(filePath, 'SPM')
clear SPM


%% VB Model Specification
% See SPM12 manual for full explanation of input measures
spm('defaults', 'EEG');
spm_jobman('initcfg');


matlabbatch{1}.spm.stats.fmri_est.spmmat = {designDirBMS};
%Select the SPM.mat file that contains the design specification.
matlabbatch{1}.spm.stats.fmri_est.write_residuals = 0;
% 0 means images are not written to disk; 1 means they are
matlabbatch{1}.spm.stats.fmri_est.method.Bayesian.space.slices.numbers = 1:91;
%1:number of time slices (an x-by-1 array must be entered)
matlabbatch{1}.spm.stats.fmri_est.method.Bayesian.space.slices.block_type = 'Slices';
% enter the block type i.e. "Slices" or "Subvolumes." - here, we use 'Slices'
matlabbatch{1}.spm.stats.fmri_est.method.Bayesian.signal = 'Global';
% Global Shrinkage prior. As explained in SPM12 help or manual
matlabbatch{1}.spm.stats.fmri_est.method.Bayesian.ARP = 3;
% An  AR  model order of 3 is the default. Cardiac and respiratory artifacts are
% periodic  in  nature  and  therefore  require  an  AR  order of at least 2. In
% previous  work,  voxel-wise selection of the optimal model order showed that a
% value of 3 was the highest order required.
matlabbatch{1}.spm.stats.fmri_est.method.Bayesian.noise.UGL = 1;
% Unweighted  graph-Laplacian.  This  is the default option.
matlabbatch{1}.spm.stats.fmri_est.method.Bayesian.LogEv = 'Yes';
```

```
        % Important: Log evidence map - Computes  the  log evidence for each voxel.
        matlabbatch{1}.spm.stats.fmri_est.method.Bayesian.anova.first = 'No';
        % ANOVA first-level implemented using Bayesian model comparison.
        % Computationally demanding: recommended option is therefore NO.
        matlabbatch{1}.spm.stats.fmri_est.method.Bayesian.anova.second = 'No';
        % ANOVA second-level. Tells SPM to automatically generate the simple contrasts
        % necessary  to produce the contrast images for a second-level (between-subject) ANOVA
        matlabbatch{1}.spm.stats.fmri_est.method.Bayesian.gcon = struct('name', {}, 'convec', {});
        % Name contrast vector: contrasts  used to generate PPMs which characterise effect sizes at
        % each voxel.
        spm_jobman('serial',matlabbatch);
        clear matlabbatch
    end
end
```

BMS2b_Source_NaNtoZeros.m

```
%% Script for Replacing NaNs with Zeros in Source LogEv.nii files
% Before running the final script (BMS3) when performing source BMS steps,
% use this script to replace non-number values (NaNs) with zeros in the
% LogEv.nii files that were created in BMS2_ModelSpec_VB.m script

% This way, the sparseness of the data does not prevent PPMs from being
% able to be made in the final BMS script.

% Requirements for this script:
```

% - SPM12 installation

% - LogEv.nii files created from the BMS2_ModelSpec_VB.m step

% We used data from an auditory oddball paradigm (as described in

% Garrido et al., 2017), obtained from the Queensland Brain Institute,

% Australia, using a 64 channel EEG Biosemi system. For the raw data,

% please see: https://figshare.com/s/1ef6dd4bbdd4059e3891 and for the

% preprocessed data, please see: https://figshare.com/s/c6e1f9120763c43e6031

% For further information on data collection and on this analysis method

% see: Garrido, M., Rowe, E., Halasz, V., & Mattingley, J. (2017).

% Bayesian mapping reveals that attention boosts neural responses to

% predicted and unpredicted stimuli. Cerebral Cortex, 1-12.

% DOI: 10.1093/cercor/bhx087

% If you use these scripts, please cite:

% Harris C.D., Rowe, E.G., Randeniya, R. and Garrido, M.I. (2018).

% Bayesian Model Selection Maps for group studies using M/EEG data.

% Scripts written by Elise Rowe, July 2016.

% Further changes made by Clare Harris and Elise Rowe, April 2017.

% Please note that these scripts have been optimised for MATLAB 2016b.

%%
%%%%%%%%%%%%%%%%%%%%%%%%%%%

```
%% Replace NaNs in the source LogEv files with zeros
PNo = 1:20; % Enter range of participant numbers in ascending order
filename = ['LogEv.nii']; % filename for log evidence images

for pp = 1:length(PNo)

   participantNo = PNo(pp);

   for model = 1:2 %enter the number of models being compared
      cd(['C:\FolderExample1\SubfolderExample1\P' num2str(participantNo) '\BMS_Model'
num2str(model) '\'])
      %Above, write the directory where the LogEv.nii files are stored.

      img = spm_vol(filename);
      img_data = spm_read_vols(img);

      for i = 1:size(img_data)
         img_data(isnan(img_data)) = 0; % replace all NaNs within the image with '0's
         img.fname = 'nLogEv.nii'; % save it as a new filename, and remember to edit
         % BMS script 3 (BMS2_PPMs.m) so that the LogEv filename matches
         spm_write_vol(img, img_data);
      end
   end
end
```

BMS3_PPMs.m

```
%% PPM Creation Script
% This script creates posterior probability maps (PPMs) and exceedance
% probability maps (EPMs); please see line 72 if you want PPMs only.
% One PPM is created for each model that was defined and estimated in
% the previous script (BMS 2). PPMs combine the log evidence across all
% participants in the dataset and are used to determine the best model.

% Requirements for this script:
% - SPM12 installation
% - Log Evidence (logEv.nii) images per participant and model from
%    completed BMS2 script (spatiotemporal) and LogEv.nii files AFTER
%    replacement of NaNs using the BMS2b script (source)
% - the correct spm_spm_vb.m script saved in the SPM12 folder
%    either for source or spatiotemporal EEG analyses (see the corresponding
%    instructions in this GitHub respository)

% We used data from an auditory oddball paradigm (as described in
% Garrido et al., 2017), obtained from the Queensland Brain Institute,
% Australia, using a 64 channel EEG Biosemi system. For the raw data,
% please see: https://figshare.com/s/1ef6dd4bbdd4059e3891 and for the
% preprocessed data, please see: https://figshare.com/s/c6e1f9120763c43e6031

% For further information on data collection and on this analysis method
% see: Garrido, M., Rowe, E., Halasz, V., & Mattingley, J. (2017).
```

```
% Bayesian mapping reveals that attention boosts neural responses to
% predicted and unpredicted stimuli. Cerebral Cortex, 1-12.
% DOI: 10.1093/cercor/bhx087

% If you use these scripts, please cite:
% Harris C.D., Rowe, E.G., Randeniya, R. and Garrido, M.I. (2018).
% Bayesian Model Selection Maps for group studies using M/EEG data.

% Scripts written by Elise Rowe, July 2016.
% Further changes made by Clare Harris and Elise Rowe, April 2017.

% Please note that these scripts have been optimised for MATLAB 2016b.

% NOTE: Set 'showModel' value for specific model results to visualise.
% Also set: PP, lovEvFilename & modelPath prior to running scripts.

%%%%%%%%%%%%%%%%%%%%%%%%%%%%%%%%%%%%%%%%%%%%%%%%
%%%%%%%%%%%%%%%%%%%%%%%%%%%

%% Start BMS 3 Process
clear all
spm('defaults', 'EEG');

PP = [1:20]; % Enter range of participant numbers in ascending order
logEvFilename = ['nLogEv']; % Change this depending on spatiotemporal or source analysis
```

```
spm_jobman('initcfg');


%% BMS at Second Level for Posterior Probability Maps

matlabbatch{1}.spm.stats.bms_map.inference.dir = {'C:\FolderExample1\SubfolderExample1\'};

% Write BMS files to the location specified above


for pNo = 1:length(PP)

   usePNo = PP(pNo);


   for model=1:2 % Change this to reflect the number of models considered

      matlabbatch{1}.spm.stats.bms_map.inference.sess_map{pNo}.mod_map{model,1} = ...

         ['C:\FolderExample1\SubfolderExample1\P' num2str(usePNo) '\BMS_Model'
num2str(model) '\' num2str(logEvFilename) '.nii,1'];

         % Change the file name to LogEv.nii when performing spatiotemporal BMS.


   end

end


matlabbatch{1}.spm.stats.bms_map.inference.mod_name = {

                                          'Model1'

                                          'Model2'

                                          };

% Above, name your models (you can enter more than 2)

% For an explanation of Model1 (Opposition Model) and Model2

% (Interaction Model) please see Garrido et al., 2017.

matlabbatch{1}.spm.stats.bms_map.inference.method_maps = 'RFX'; %RFX = random effects, FFX
= fixed effects
```

matlabbatch{1}.spm.stats.bms_map.inference.out_file = 1; % 0 = output PPMs only, 1 = output PPMs and EPMs

matlabbatch{1}.spm.stats.bms_map.inference.mask = {''}; % blank for no mask

spm_jobman('serial',matlabbatch);

clear matlabbatch

%% Show PPM for one model (at a time)

showModel = 'Model1'; % Show results for this model - change as desired

modelPath = ['C:\FolderExample1\SubfolderExample1\' num2str(showModel) '_model_xppm.nii,1'];

% The above line sets the path for model results.

spm('defaults', 'EEG');

spm_jobman('initcfg');

matlabbatch{1}.spm.stats.bms_map.results.file = {'C:\FolderExample1\SubfolderExample1\BMS.mat'};

matlabbatch{1}.spm.stats.bms_map.results.img = {modelPath};

matlabbatch{1}.spm.stats.bms_map.results.thres = 0.75; % set probability of winning model here

matlabbatch{1}.spm.stats.bms_map.results.k = [1 1]; % extend results this many voxels

matlabbatch{1}.spm.stats.bms_map.results.scale = [1]; % 1 = log; 0 = none; [] = serial mode

spm_jobman('serial',matlabbatch);

This is the end of the scripts. For the live copies, including any potential future updates, please see the scripts stored on Github (https://github.com/ClareDiane/BMS4EEG).
